# A novel root hair mutant, *srh1*, affects root hair elongation and reactive oxygen species levels in wheat

**DOI:** 10.3389/fpls.2024.1490502

**Published:** 2024-10-30

**Authors:** Ian Tsang, Pauline Thomelin, Eric S. Ober, Stephen Rawsthorne, Jonathan A. Atkinson, Darren M. Wells, Lawrence Percival-Alwyn, Fiona J. Leigh, James Cockram

**Affiliations:** ^1^ Plant Genetics Department, NIAB, Cambridge, United Kingdom; ^2^ Department of Plant Science, University of Nottingham, Nottingham, United Kingdom; ^3^ The Morley Agricultural Foundation, Wymondham, United Kingdom

**Keywords:** temperate cereal species, sustainable crop production, root system architecture, exome capture, RNA sequencing (RNA-Seq), differential gene expression (DEG)

## Abstract

**Background:**

Root hairs are single-celled projections on root surfaces, critical for water and nutrient uptake. Here, we describe the first short root hair mutant in wheat (*Triticum aestivum* L.), identified in a mutagenized population and termed here short root hair 1 (*srh1*).

**Results:**

While the *srh1* mutant can initiate root hair bulges, lack of subsequent extension results in very short root hairs. Due to its semi-dominant nature, heterozygous lines displayed intermediate root hair lengths compared to wild-type. Bulked segregant analysis in a BC_1_F_3_ segregating population genotyped via exome capture sequencing localized the genetic control of this mutant to a region on the long arm of chromosome 3A. Via RNA sequencing and bioinformatic analysis, we identified two promising candidate genes. The first was a respiratory burst oxidase homolog (RBOH) encoding gene *TaNOX3-A*, orthologous to RBOH genes controlling root hair elongation in rice (*OsNOX3*) and maize (*ZmRTH5*), that carries a missense mutation in a conserved region of the predicted protein. RBOHs are membrane bound proteins that produce reactive oxygen species (ROS) which trigger cell wall extensibility, allowing subsequent root hair elongation. Notably, reduced ROS levels were observed in *srh1* root hair bulges compared to wild-type. The second candidate was the calreticulin-3 encoding gene *TaCRT3-A*, located within the wider *srh1* interval and whose expression was significantly downregulated in *srh1* root tissues.

**Conclusions:**

The identification of a major effect gene controlling wheat root hair morphology provides an entry point for future optimization of root hair architecture best suited to future agricultural environments.

## Highlights

- We describe the wheat root hair mutant, *short root hair 1* (*srh1*). Mapped to chromosome 3A, *srh1* reduces reactive oxygen species production and affects root hair elongation, but not initiation.

## Introduction

Hexaploid bread wheat (*Triticum aestivum* L.) is one of the world’s most important crops, and accounts for 20% of the global human calorie intake (FAO, 2022). Whilst not under direct selection by breeders, there is indication that across wheat breeding history, root hairs have been decreasing in size. With the impacts of climate change and focus on reduced input agriculture, current wheat root hair morphology and function may be sub-optimal for future agricultural environments. Thus, root hairs serve as a targetable trait for plant breeding with which to help optimize wheat yields and resilience, as well as reducing the environmental footprint of wheat cultivation (Tsang et al., 2024).

Root hair development in plants can be broadly split into four major stages (Grierson et al., 2014). The first - cell fate determination - regulates the fate of epidermal cells on the emerging primary root (radicle), where cells can differentiate into either a hair cell (trichoblast) or a non-hair cell (atrichoblast). The second stage involves bulge initiation, where the cell wall forms a bulge at the base of an emerging root hair (Grierson et al., 2014). Elongation of the bulge occurs in the third stage via a process known as tip growth, until the root hair reaches full length at maturity during the fourth and final stage (Grierson et al., 2014).

In the model plant *Arabidopsis* (*Arabidopsis thaliana* L.), the four stages of root hair development are each regulated by a complex network of genes and transcription factors (Balcerowicz et al., 2015). Across the world’s major cereal species, some of these genes have been characterized (recently reviewed by Tsang et al., 2024). For example, in rice (*Oryza sativa* L.), *OsRHL1* encodes a basic helix-loop-helix (bHLH) transcription factor regulating root hair length (Ding et al., 2009). *OsEXPA8* and *OsEXPB5* encode expansins that facilitate cell wall extension to promote subsequent root hair elongation (Ma et al., 2013). *OsRBOH5* encodes a reactive oxygen species (ROS) producing enzyme, catalyzing production of free oxygen radicals to aid cell wall extensibility and root hair bulge formation (Wang et al., 2018). Examples of characterized maize (*Zea mays* L.) root hair genes include *ZmRTH3*, which encodes a COBRA-like protein that functions as a Glycosylphosphatidylinositol (GPI) anchor, organizing synthesized cellulose bundles from cellulose synthases for root hair extension (Hochholdinger et al., 2008), and *ZmRTH6*, which encodes a cellulose synthase (Li et al., 2016). Despite the global importance of wheat, and the central role root hairs play in water and nutrient acquisition, current information on the genetic control of wheat root hair development is very limited (Han et al., 2016, Han et al., 2017; Zeng et al., 2024). While no wheat genes controlling root hair development have been positionally cloned to date, putative genes acting within the pathway have begun to be identified. Some of the first identified were the wheat Class II bHLH transcription factors *TaRSL2* and *TaRSL4*, which share sequence homology with the *Arabidopsis* root hair genes *ROOT HAIR DEFECTIVE-SIX LIKE 2* (*AtRSL2*) and *AtRSL4* (Ma et al., 2013, Han et al., 2016, Han et al., 2017). Based on the observation that increase in root hair length in wheat species with increased ploidy is associated with higher *TaRSL2* and *TaRSL4* expression (Han et al., 2016), and that ectopic expression of *TaRSL2* in *Arabidopsis* increased root hair length, these wheat genes have been hypothesized to function in a similar manner to their *Arabidopsis* homologs in root hair development. Historically, the size and complexity of the 17Gb hexaploid wheat genome has hindered progress in understanding the genetic control of wheat traits, including root hair development. However, recent advances including the availability of genome assemblies (The International Wheat Genome Sequencing Consortium (IWGSC) et al., 2018; Walkowiak et al., 2020) and resources such as Ethyl methanesulfonate (EMS) mutagenized Targeting Induced Local Lesions IN Genomes (TILLING) populations (Krasileva et al., 2017), have begun lowering the barriers to genetic locus and gene identification in wheat. These resources have been recently used to identify a candidate gene (*stumpy*) on chromosome 7B encoding a putative transmembrane protein associated with calcium ion (Ca^2+^) transport, mutation in which is thought to lead to increased root hair length when grown in high Ca^2+^ soils (Zeng et al., 2024).

In this paper, we report the discovery of a semi-dominant wheat mutant controlling root hair extension, which we term *short root hair 1* (*srh1*). We identified a candidate gene (*TaNOX3-A)* that is located close to the peak of the *srh1* genetic interval, is orthologous to genes known to control root hair length in maize and rice, and which contains a missense mutation in a conserved region in the predicted protein. Additionally, within the wider *srh1* genetic interval, we identified a wheat Calreticulin-3 gene that was highly down-regulated in *srh1* roots compared to wild-type, indicating a likely role of calreticulin in root hair development.

## Materials and methods

### Identification of the *srh1* mutant

The wheat *srh1* mutant was identified by phenotypic screening of a wheat cv. Cadenza TILLING population (Krasileva et al., 2017) using M_4_ generation seed sourced from the SeedStor, United Kingdom (UK) (https://www.seedstor.ac.uk). The mutation was identified visually in Cadenza TILLING line 1714, and M_4_ seedlings with the *srh1* phenotype were then selfed to the M_5_ generation. M_5_ seed was phenotyped to check for phenotypic segregation, and two short root hair M_5_ lines derived from a single M_4_ plant were back-crossed to wild-type Cadenza to form the backcross-1 first filial (BC_1_F_1_) generation, and then selfed to yield BC_1_F_2_ and subsequent BC_1_F_3_ generations. Selected BC_1_F_2_ individuals shown phenotypically to be homogyzous for the short root hair phenotype were crossed to wild-type to generate a BC_2_F_1_, and selfed to generate BC_2_F_2_ and BC_2_F_3_ generations.

### Root phenotyping

For root hair phenotypic measurements, BC_1_F_2_ seedlings were surface sterilized with 5% (v/v) bleach (sodium hypochlorite) and cold-treated at 4°C for 5 days in the dark. Seedlings were sown on agar (1%, pH7) plates which were sealed with micropore tape and left to germinate at room temperature for 3 days in the dark. Three-day old seedlings were imaged under a stereo-microscope (Leica MZ10F, Leica, Germany) with a Leica DFC 295 Camera (Leica, Germany). Measurement of root hair length (RHL), seminal root length (SRL), primary root length (PRL) and total root length (TRL) using the images obtained was performed using the software, FIJI (Schindelin et al., 2012). To capture images for root hair quantification, a Raspberry Pi HQ camera (Pi Hut, UK) with an attached macro lens (Pimoroni, UK) was used. Representative root hairs that grew flat on the agar surface were measured for 10 individuals of each genotype using FIJI (Schindelin et al., 2012)

To asses root hair phenotype in soil-grown plants, BC_2_F_4_ *srh1* and wild-type segregant lines were grown in 12cm x 12cm petri dishes filled with moist soil: silty clay loam in the Hornbeam series (chromic endostagnic Luvisol) with pH 6.1, P index 1(12.4mg L-1 plant available Olsen P), K index 1 (94mg L-1 available K) and Mg index 2 (66mg L-1 available Mg). Six seedlings of each line were grown for seven days in a controlled environment chamber (18hr light at 20°C, 6hr dark at 16°C), after which roots were imaged using a Leica DM500 microscope and visually assessed for the presence/absence of root hairs.

### Cryo-SEM

Wild-type Cadenza and BC_1_F_2_ *srh1* mutants were sown on 1.5% agar medium for 5-7 days. Using a sterile scalpel, 1cm root segments from the root hair differentiation zone were excised and mounted on a stub covered with a glue/graphite mixture. Samples were immersed in liquid nitrogen and transferred to a vacuum chamber to be coated in platinum. Two sets of samples were produced: for batch one, water was removed via sublimation and shadowed with 7nm platinum; for batch two, samples were fractured and shadowed with 7nm platinum. Cryo-scanning electron microscopy (Cryo-SEM) imaging was carried out using a Zeiss EVO HD15 (Zeiss, Oberkochen, Germany). To calculate root hair density, measurements were taken from images captured under Cryo-SEM. Ten independent, randomly assigned field of views were each used to score root hair density in wild-type and *srh1* root samples.

### Bulked segregant analysis and exome capture

#### Library prep

Three-day old seedlings from the BC_1_F_3_ generation were phenotyped as homozygous mutant (short root hair) or homozygous wild-type (wild-type root hair length). The wild-type bulk contained 55 seedlings while the *srh1* bulk contained 63 seedlings. Seedling root DNA from each bulk was extracted using a QIAGEN DNeasy Plant Mini Kit, following the manufacturer’s instructions. Exome capture was carried out via subcontract to Earlham Institute (UK): the sequencing library was constructed using a KAPA HTP kit (Roche) and were hybridized with exome capture baits (Gardiner et al., 2019). Fragments were sequenced using a 300 SP flow cell on a NovaSeq 6000 sequencing system (Illumina), generating 150bp paired-end reads.

#### Exome capture sequence alignment and bulked frequency ratio

Quality control of the reads before and after adapter removal was carried out using FastQC (Babraham Bioinformatics). Illumina adapter sequences were trimmed using Trim Galore (Babraham Bioinformatics). Reads with a Phred quality score of <25 and length <100bp were discarded. Trimmed reads were aligned to the indexed wheat reference genome of cv. Chinese Spring (RefSeq v1.1; IWGSC, 2018) using Burrows Wheeler Aligner (BWA) (Li and Durbin, 2009). Alignment files were sorted using Samtools (Danecek et al., 2021) prior to marking polymerase chain reaction (PCR) duplicates for removal using Picard MarkDuplicates (http://broadinstitute.github.io/picard). Alignments were then filtered using Samtools (Danecek et al., 2021) to retain properly paired reads while removing non-primary alignments and PCR duplicates. Variant calling was carried out using BCFtools mpileup (Danecek et al., 2021), discarding alignments with a minimum mapping quality < 30, and a coefficient for downgrading mapping quality of 50 was used, as recommended for BWA alignments.

To assess sequencing depths at gene regions, Samtools depth was used to construct a table of depths across genic regions +/- 50% of gene lengths for each bulk (**Supplementary Figure S2**).

Bulked allele frequencies were calculated as follows (as previously described by Martinez et al., 2020):


1 BAF=AA/RD

where BAF = bulked allele frequency, AA = alternate allele read depth, RD = total read depth. Bulked frequency ratio (BFR) was then calculated as:


2 $$BFR=BF_{srh1}-BF_{WT}$$

Bulked frequency ratios (BFRs) between the wild-type and *srh1* bulk were calculated from the variant calls after applying the following filters in VCFtools (Danecek et al., 2011): a minimum depth of 5, a maximum depth of 200 and exclusion of InDels and missing genotypes. BFR values were then computed using a bespoke python script across a 27.5Mb jumping window with a 1.5Mb jump. The outputted table was then visualized using CIRCOS (Krzywinski et al., 2009).

#### Variant effects

Variant annotation was carried out using SnpEff and Ensembl’s Variant Effect Predictor (VEP) (Cingolani et al., 2012; McLaren et al., 2016). Filtering of variants was carried out to discard single nucleotide polymorphisms (SNPs) with a quality score of ≤ 50 and a combined sample depth of ≤ 20. Non-exome variants were removed, and the genes containing variants were then filtered for those showing expression in root tissues using publicly available expression databases (expVIP, Borrill et al., 2016). Variant Sorting Intolerant From Tolerant (SIFT) scores were then predicted using VEP, retaining only non-synonymous variants with a score of ≤ 0.05. The remaining variants and the flanking amino acid regions surrounding the variant of interest were aligned against plant ‘orthologs’ as identified in Ensembl Plants (https://plants.ensembl.org/index.html), and filtered based on sequence homology (**Supplementary Figure S3**). Variants with an amino acid conservation of ≥ 70% across all plant orthologs were retained.

### Reactive oxygen species quantification

For quantification of ROS levels in root hairs, seedlings from the BC_2_F_3_ generation were germinated on filter paper in the dark for 3 days at room temperature. 2^′^, 7^′^-Dichlorodihydrofluorescein diacetate (H_2_DCF-DA) incubation solution was prepared fresh as follows: 1mg of H_2_DCF-DA was dissolved in 1ml of DMSO, and the mixture diluted to 200ml with dH_2_O (Nestler et al., 2014). The seedlings were submerged in the H_2_DCF-DA solution for 20 minutes and rinsed twice in Phosphate Buffered Saline (PBS) buffer (Sigma Aldrich, P4417), then individually placed in cover glass bottomed chambers (Nunc LabTek II 1 Well Cover Glass, Thermo Fisher Ltd.). Roots were sandwiched between the glass and nutrient agar to force roots to lay flat. The chambers were imaged under a Leica SP8 Confocal Microscope (Leica, Wetzlar, Germany). Excitation was performed using an argon laser at 488nm and captured between 500-540nm. Z-projections were captured across the visible epidermal cell layers and maximal Z-projections were constructed in FIJI (Schindelin et al., 2012).

To quantify fluorescence intensity at root hair tips, a region of interest was first created around a 20*µ*m section of the root hair tip in FIJI (Schindelin et al., 2012). Pixel thresholding was performed in the region of interest containing the root hair tip across all Z-stacks containing the same root hair. Thresholding boundaries for minimum and maximum pixel intensity were set at 10 and 255, respectively. The pixel value for each root hair was calculated as a mean across Z-stacks.

### Sanger sequencing of *TaNOX3-A*

To confirm the presence of the candidate SNP in *TaNOX3-A*, mutant and wild-type individuals from the BC_2_F_4_ generation were grown in soil in a controlled growth environment under 16hr day; 20°C, 6hr night; 18°C conditions. First leaf tissue was harvested from mutant and wild-type plants after 2 weeks, and DNA was extracted from the leaves following the Tanksley DNA extraction protocol (Fulton et al., 1995). Phusion HF DNA Polymerase (Thermo Fisher Ltd) was used to PCR amplify the DNA fragment containing the target SNP with the following cycle (98°C 30s; 40 cycles of 98°C 10s, 66.5°C 30s, 72°C 15s; 72°C 10min) using homoeologue-specific primers (F-GAGGTGCGGCAGTAATGATAT, R-AGTTGCCTGAACTGACCTTC). The PCR products were size checked using electrophoresis across a 1% agarose gel and were subsequently excised from the gel under UV illumination, and extracted using the Thermo Fisher GeneJet Gel Extraction Kit (Thermo Fisher Ltd.) following manufacturer guidelines. DNA Sanger sequencing was outsourced to Source Bioscience, UK, and the sequence chromatograms were visualized using Geneious (https://www.geneious.com/).

### RNA-seq

#### Library Prep

RNA extracted from 3-day old wild-type and *srh1* seedlings from the BC_2_F_3_ generation were used to construct libraries for RNA sequencing (RNA-seq). The seedlings were germinated on filter paper at room temperature and grouped based on root hair phenotype. RNA was extracted using a QIAGEN RNeasy Plant Mini Kit from the seminal roots of 30 wild-type and 30 *srh1* individual seedlings across three biological replicates per genotype. For each root, RNA was extracted from two regions: the elongation zone (∼ 1cm from root tip) and the mature zone (∼ 2-3cm above the root tip). Illumina libraries were prepared and sequenced via subcontract to Novogene (Cambridge, UK) using an Illumina NovaSeq 6000 S4 flowcell generating 150bp paired-end reads.

#### Alignment and read counts

FastQC (Babraham Bioinformatics) was used to assess read quality before and after adapter trimming. Adapter trimming was performed using Trim Galore (Babraham Bioinformatics). RNA-seq reads were aligned to the indexed wheat reference genome (RefSeq v1.0; IWGSC, 2018) using Hisat2 (Kim et al., 2019). Aligned reads were sorted using Samtools (Danecek et al., 2021). FeatureCounts (Liao et al., 2014) was used to output a counts matrix from the BAM files and gene models (RefSeq v1.1, IWGSC, 2018).

#### Differential gene expression

The RNA-seq data was used to determine differentially expressed genes (DEGs) between the following treatments: overall root tissue between *srh1* and wild-type; mature root tissue between *srh1* and wild-type, tip tissue between *srh1* and wild-type; and mature tissue versus tip tissue for *srh1* and wild-type. Differential gene expression analysis was conducted in RStudio (Version 4.3.2, RStudio Team, 2020) using r/DESeq2 (Love et al., 2014). Quality control, count normalization and clustering were performed in DESeq2, while shrinkage of Log_2_Fold Change (Log_2_FC) was carried out using lfcShrink() (Zhu et al., 2019). To yield manageable lists of DEGs, significance and Log_2_FC thresholds were adjusted according to each comparison. For differential gene expression analysis in overall root tissues, genes were deemed significantly differentially expressed with a false discovery rate (FDR) ≤ 0.01 and Log_2_FC ≥ abs(1.5). Across mature root tissues, thresholds for FDR and Log_2_FC were ≤ 0.001 and ≥ abs(2), respectively. For root tip tissues, thresholds for FDR and Log_2_FC were 0.01 and abs(1.5), respectively. Volcano plots were created using r/EnhancedVolcano (Bilghe et al., 2023).

#### Functional annotation of differentially expressed genes

To identify functional annotation terms associated with candidate genes, the IWGSC gene model IDs were searched against the gene functional annotations (IWGSC, 2018). Traits putatively associated with DEGs were predicted using KnetMiner (Hassani-Pak et al., 2021).

#### Homology and protein modeling

Wheat homologues of genes originally characterized in other plant species were identified using coding regions (CDS) as queries for BLASTn analysis of the wheat reference genome (RefSeq v1.0) in Ensembl Plants (Yates et al., 2022). For analysis of the predicted proteins encoded by homologous genes across many plant species, gene/protein lists were exported from Ensembl Plants using the “orthologues” function. Plots of amino acid conservation were generated using WebLogo (Crooks et al., 2004). To clarify comparison of homologous and/or orthologous genes between species, we prefix gene names with genus and species initials.

Protein modeling was carried out using SWISS-MODEL (https://swissmodel.expasy.org/). Protein sequences (in FASTA format) were downloaded from Ensembl Plants (https://plants.ensembl.org/index.html), protein models were constructed using the ‘Build Model’ option.

## Results

### The semi-dominant *short root hair 1* mutant controls root hair elongation

**Figure 1:**
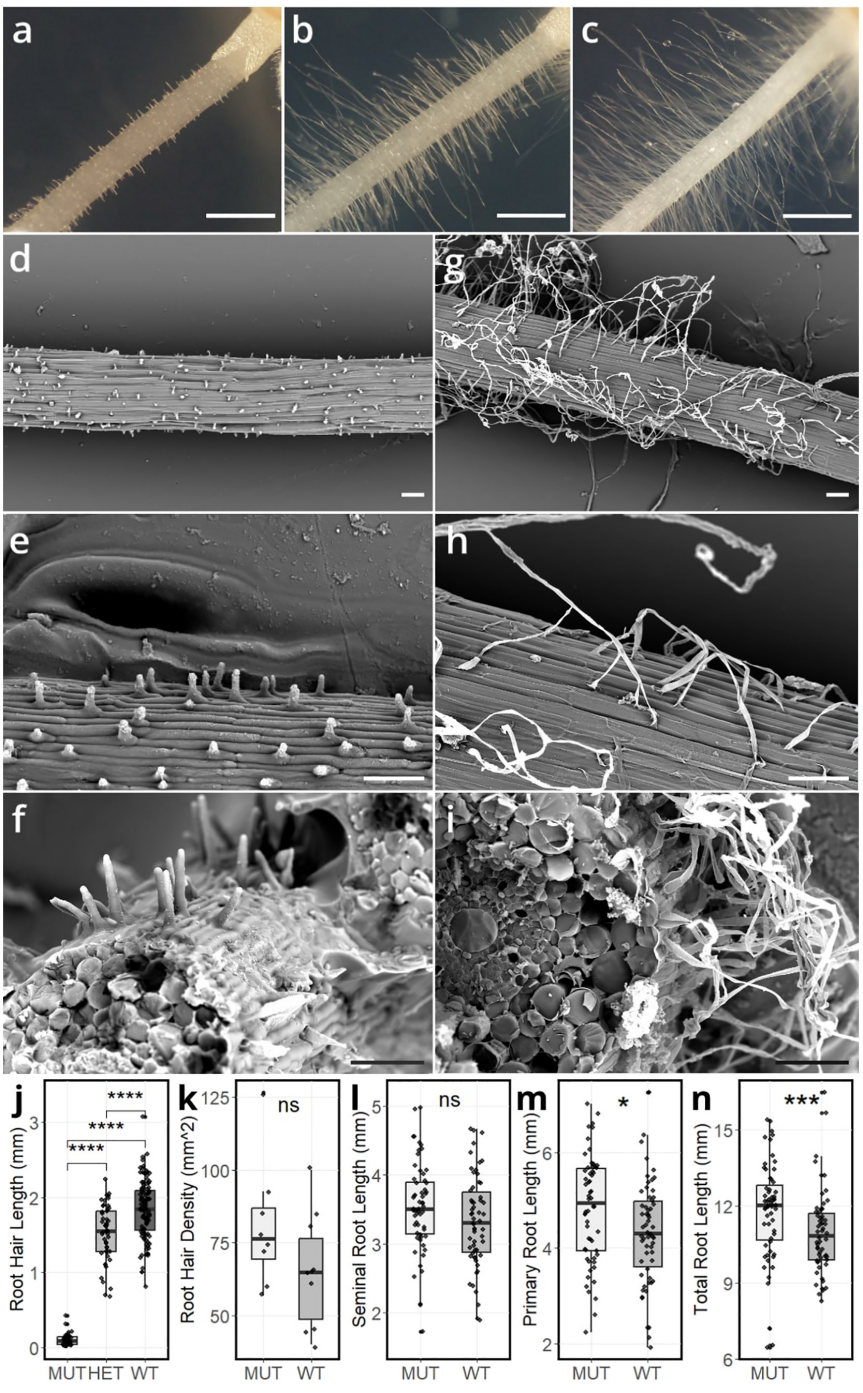
Seminal roots depicting root hair morphology in 3-day old BC_1_F_2_ seedlings segregating for *srh1* and wild-type phenotype: **(A)**: *srh1* mutant **(B)**, heterozygous **(C)** wild-type. Cryo-SEM images of the *srh1* mutant **(D–F)** and wild-type **(G–I)** across increasing magnifications. Scale bars: **(A–C)** 1mm, **(D–I)** 100*µ*m. **(J–N)**: Root morphological differences between *srh1*, heterozygous and wild-type. **(J)**: Root hair length on seminal roots. **(K)**: Root hair density. **(L)**: Seminal root length. **(M)**: Primary root length, **(N)**: Total root length (primary root + seminal roots). Significance as determined by T-test: * p <0.05, *** p <0.001, **** p <0.0001, Ns – not significant.

The disrupted root hair phenotype of the *srh1* mutant was initially identified visually at the seedling stage in the Cadenza TILLING line 1714 during a screen of the population. Examination of segregating BC_1_F_2_ lines under a microscope showed that very short root hairs were found on the root surface of both primary and seminal roots in the mutant (e.g. **Figure 1A**). High resolution cryo-SEM imaging revealed that root hairs successfully initiate, but fail to extend in mutant lines compared to wild-type (**Figures 1D, E, G, H**). In soil, the *srh1* mutant also displays a vestigial root hair phenotype, while the wild-type segregant produces normal root hairs (**Supplementary Figure S7**). Root hair density was not significantly different in the mutant compared to wild-type (**Figure 1K**). No visual differences in trichoblast lengths were observed between *srh1* and wild-type. Cross-section cryo-SEM images (**Figures 1F, I**) did not reveal any major root tissue structural differences between genotypes. Together, these data indicate that while root hair initiation occurs in the mutant, root hair tip growth is impeded. Accordingly, we named the mutant *short root hair 1* (*srh1*).

Analysis of 266 BC_1_F_2_ seedlings (three days old) found root hair length to segregate into three visually distinct (**Figure 1J**) phenotypic categories: 62 wild-type (long root hairs), 141 heterozygous (medium length root hairs) and 63 *srh1* (no visible root hairs) (**Figures 1A-C**), consistent with a 1:2:1 segregation ratio, indicating *srh1* is controlled in a Mendelian fashion via a single semi-dominant genetic locus (*χ* ^2^ = 0.518, p<0.05). Primary root length at seedling stage in the *srh1* mutant was significantly longer (4.82 ± 0.143 cm, p<0.05) than wild-type (4.31 ± 0.14 cm), as was *srh1* total root length (11.8 ± 0.24 cm, p<0.001) when compared to wild-type (11 ± 0.24 cm). No significant differences in seminal root length, number or root hair density were observed between mutant and wild-type (**Figures 1J-N**).

### ROS production is impaired in *srh1*

**Figure 2:**
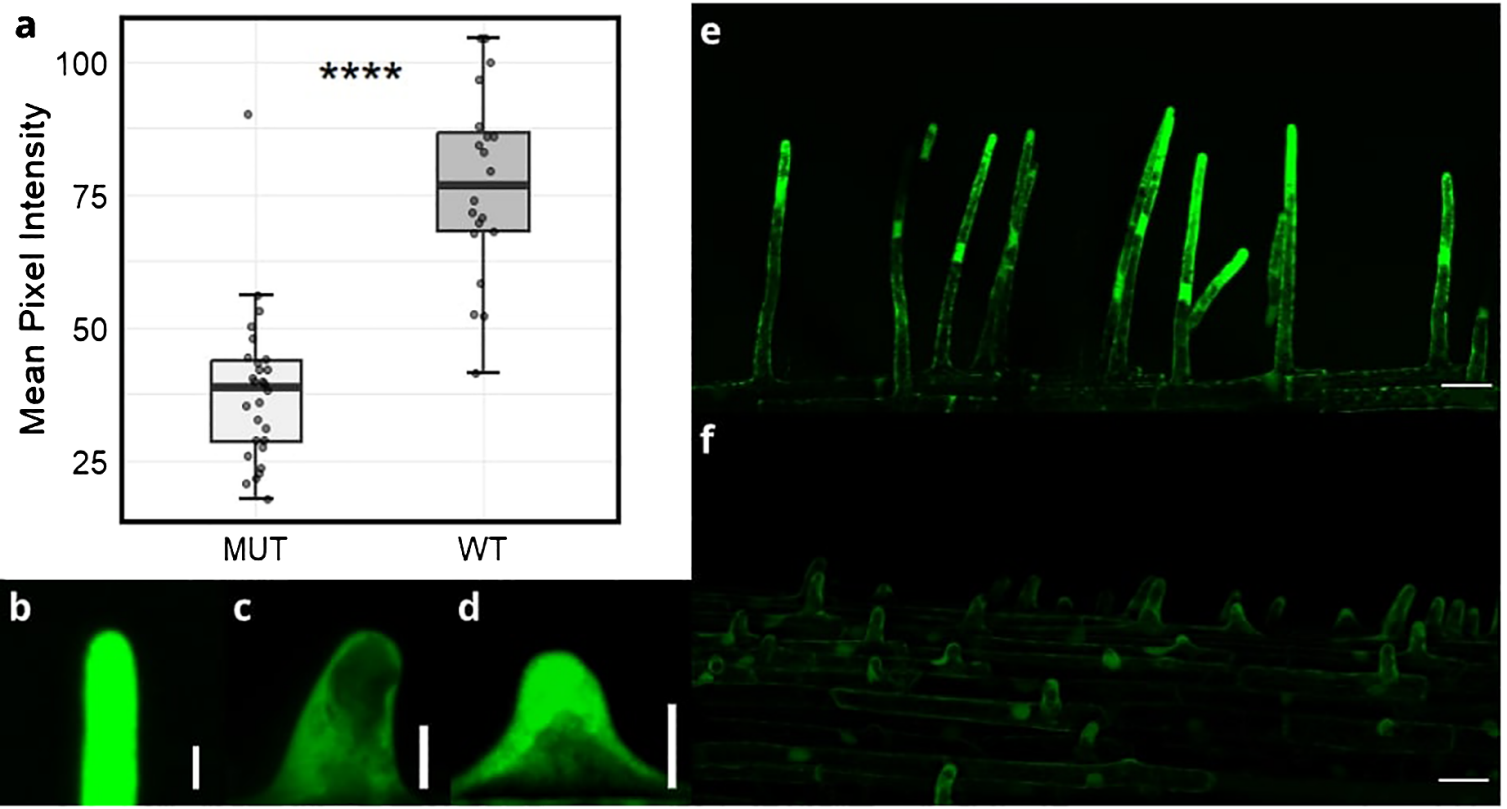
Reactive oxygen species (ROS) levels in emerging/short root hairs of the wheat *srh1* mutant (MUT) versus wild-type (WT). **(A)**: Difference in pixel intensity between root hair tips in *srh1* and wild-type BC_2_F_3_ individuals (WT: n=20, *srh1*: n=28). **(B–D)**: 20*µ*m sections of root hair tips in **(B)**: root hairs in wild-type, **(C)**: *srh1* tips, d: emerging wild-type tips. Fluorescence levels are much higher in **(E)**: wild-type hairs compared to **(F)**: *srh1*. Scale bars: **(B–D)**: 10*µ*m, **(E, F)**: 50*µ*m. Significance as determined by T-test: **** p <0.0001.

ROS production is involved in cell wall polymer cleaving - a process essential for subsequent root hair elongation. To determine whether *srh1* affected ROS levels in root hairs, we used the fluorescent dye H_2_DCF-DA to detect fluorescence in root hair tips of *srh1* and the wild-type seedlings. Mean pixel intensity in 20*µ*m segments of root hair tips showed a significant (p<0.001) decrease between the mutant and wildtype (**Figures 2A, C, D**). A high level of fluorescence was maintained in elongating root hair tips in wild-type (**Figures 2B, F**), while low fluorescence was consistent across *srh1* root hair bulges (**Figure 2E**).

### The *short root hair 1* mutant phenotype is controlled by a genetic locus on chromosome 3A

To identify the genetic locus controlling *srh1*, bulked segregant analysis was performed via exome capture of genomic DNA extracted from bulks of 55 wild-type and 63 *srh1* seedlings. Differences between allelic ratios were calculated following variant calling on the exome capture sequence alignments. At the genetic loci responsible for the *srh1* phenotype, we expect differences in the ratio of alleles between the mutant and wild-type bulks due to high linkage disequilibrium with the underlying genetic mutation(s).

**Figure 3:**
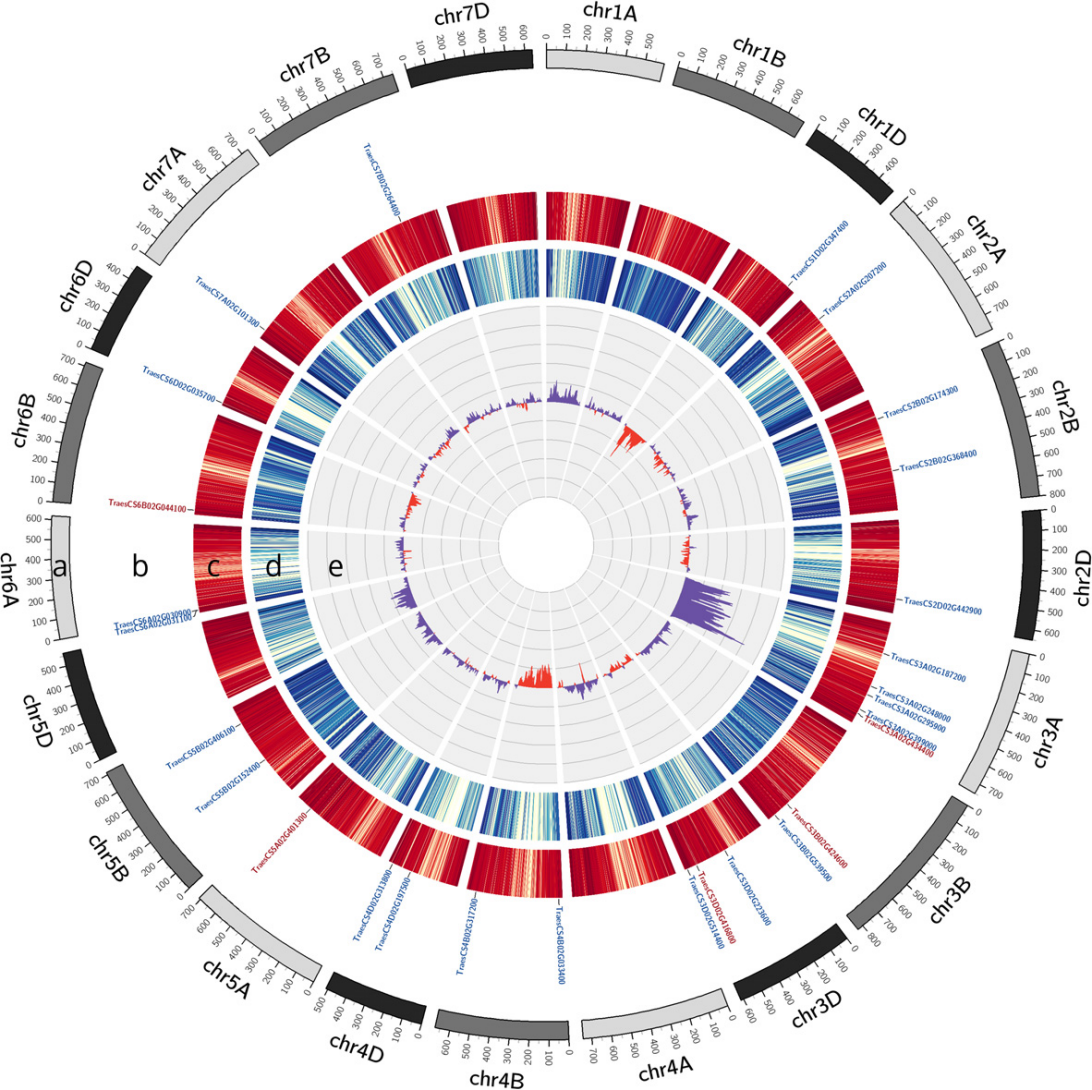
Genetic mapping of *srh1* to the long arm of chromosome 3A via bulk segregant analysis (BSA). DNA variants were identified by exome capture sequencing of BC_1_F_3_ individuals, and mapped via analysis of bulked frequency ratios (BFR) between *srh1* and wild-type bulks, aligned to the wheat reference genome. **(A)**: Wheat karyotype. **(B)**: Differentially expressed genes between *srh1* and wild-type. Down-regulated genes in red, up-regulated genes in blue (Threshold FDR ≤ 0.01 and Log_2_FC ≥ abs(1.5)). **(C)**: High confidence (HC) gene density, **(D)**: Filtered SNP density, **(E)**: BFR calculated from BF*_srh_*_1_ - BF*_WT_*.

Bulked segregant analysis located *srh1* to between 584-647Mb (defined as BFR ≥ 0.35) on the long arm of chromosome 3A (**Figure 3**). Within this interval, the highest difference in allelic ratios spanned a 22Mb interval between 595Mb - 617Mb (defined as BFR ≥ 0.5), with a maximal BFR value at ∼ 606Mb (BFR 0.66). Within this peak region, 204 of the 210 high confidence (HC) gene models in the reference genome contained associated variants assayed via exome capture. After discarding non-exome variants, we retained 39 SNPs across 33 genes. These variants were filtered for SIFT score and known expression in root tissue in public gene expression databases to identify a preliminary candidate gene list (**Table 1**). Seven SNPs across seven genes remained after filtering. Of these genes, four contained SNPs typically induced by EMS-mutagenesis (C to T, G to A), including: a predicted Glutamyl-tRNA amidotransferase (*TraesCS3A02G347600*), a predicted respiratory burst oxidase homolog (RBOH) (*TraesCS3A02G354200*), a pumilio-like protein (*TraesCS3A02G361700*) and a BEL-1 like homeodomain protein (*TraesCS3A02G363900*) (**Table 1**). The remaining three genes harbored atypical EMS-induced nucleotide substitutions, and were predicted to encode: a putative protein kinase (*TraesCS3A02G351500*), a Rop guanine nucleotide exchange factor (*TraesCS3A02G364400*) and a pentatricopeptide repeat containing protein (*TraesCS3A02G367700*).

**Table 1:** *srh1* candidate genes identified through bulked segregant analysis and exome capture.

Wheat Gene ID	EMS Variant Location in Wheat Reference Genome	SNP in Candidate Gene CDS	Resulting Amino Acid Substitution	Gene Functional Annotation	SIFT Score	Residue Conservation Across Plant Homologs
TraesCS3A02G347600*	596743515	1166 G >A	G389E	Glutamyl-tRNA amidotransferase subunit A	0	95%
*TraesCS3A02G351500*	600007728	1799 A >G	D600	Protein Kinase	0	95%
TraesCS3A02G354200*	601558511	1427 C >T	A476V	Respiratory burst oxidase-like protein	0	90%
TraesCS3A02G361700*	609947615	187 G >A	E63K	Pumilio-like protein	0	84%
TraesCS3A02G363900*	612526030	1103 C >T	P368L	BEL1-like homeodomain protein	0	94%
*TraesCS3A02G364400*	613172884	1459 A >G	S487G	Rop guanine nucleotide exchange factor	0.04	96%
*TraesCS3A02G367700*	616705229	693 G >T	E231D	Pentatricopepetide repeat-containing protein	0	86%

Candidates were identified based on filtering for genes within the peak *srh1* physical interval (595 - 617Mb) within the wheat reference genome (cv. Chinese Spring, IWGSC v1.1) which carried non-synonymous/deleterious EMS mutations, effect on predicted protein (SIFT Score), and expression in wheat root tissues (based on public gene expression datasets). *Genes harboring SNPs typically induced by EMS mutation (C >T, G >A).

The EMS induced SNPs across these seven genes all resulted in missense amino acid substitutions spanning highly conserved predicted protein domains (**Supplementary Figure S3**). The G to A DNA substitution at position 1166bp in the genomic sequence of the glutamyl-tRNA amidotransferase subunit A gene *TraesCS3A02G347600* (g.1166G>A) resulted in a glycine (G) to glutamic acid (E) amino acid substitution at residue 389 in a predicted amidase signature domain within the predicted protein (a.G389E), with the G residue having 95% conservation across 152 plant homologs. The g.1799A>G substitution in the protein kinase *TraesCS3A02G351500* resulted in an aspartic acid (D) to G amino acid substitution (a.D600Q) in a predicted protein kinase domain, with the D residue having 95% conservation across 159 plant homologs. In the RBOH gene *TraesCS3A02G354200*, the g.1427C>T mutation (**Supplementary Figure S6A**) resulted in an Alanine (A) to Valine (V) amino acid substitution (a.A476V) within a predicted ferric reductase transmembrane domain, with the A residue having 90% conservation across 157 homologs (**Supplementary Figure S6B**). The g.187G>A mutation in the pumilio-like gene *TraesCS3A02G361700* resulted in a E to Lysine (K) substitution (a.E63K). Although not located in a predicted protein domain, the E residue at position 63 had 84% conservation across 244 plant homologs. In the BEL-1 like homeodomain gene *TraesCS3A02G363900*, the g.1103C>T mutation resulted in a Proline (P) to Leucine (L) substitution (a.P368L) at a residue with 94% conservation across 185 homologs within a predicted homeobox protein domain. The g.1459A>G mutation in *TraesCS3A02G364400*, a Rop guanine nucleotide exchange factor, resulted in a serine (S) to G amino acid substitution (a.S487G). While not predicted to lie within an annotated protein domain, the S residue was 96% conserved across 24 plant homologs. The g.693G>T mutation in the pentatricopeptide repeat containing gene *TraesCS3A02G367700* resulted in an E to D substitution (a.E231D) within a pentatricopeptide repeat domain, with the E residue conserved at 86% across 119 plant homologs (**Supplementary Figure S3**). Additionally, within the wider *srh1* genetic interval, we identified a g.1077G>A substitution at in the coding sequence of *TraesCS3A02G39900* (*TaCRT3-A*), which resulted in an amino acid substitution of tryptophan (W) >STOP (a.W359STOP) (SIFT score = 0) resulting in the truncation of an entire alpha helix towards the C-terminus of the predicted protein (**Supplementary Figure S1**).

### Differentially expressed genes

To help inform candidate gene analysis, as well as identify additional genes whose expression may have been altered in the *srh1* mutant background, we analyzed RNA-seq data from root tissues extracted from two zones of primary roots (root tip and mature zone) of BC_1_F_3_ lines with either mutant *srh1* or wild-type phenotype. Across *srh1* and wild-type tissues from both root zones (i.e. considering tip and mature zone together), we identified a total of 67651 genes.

#### Overall primary root tissues

**Figure 4:**
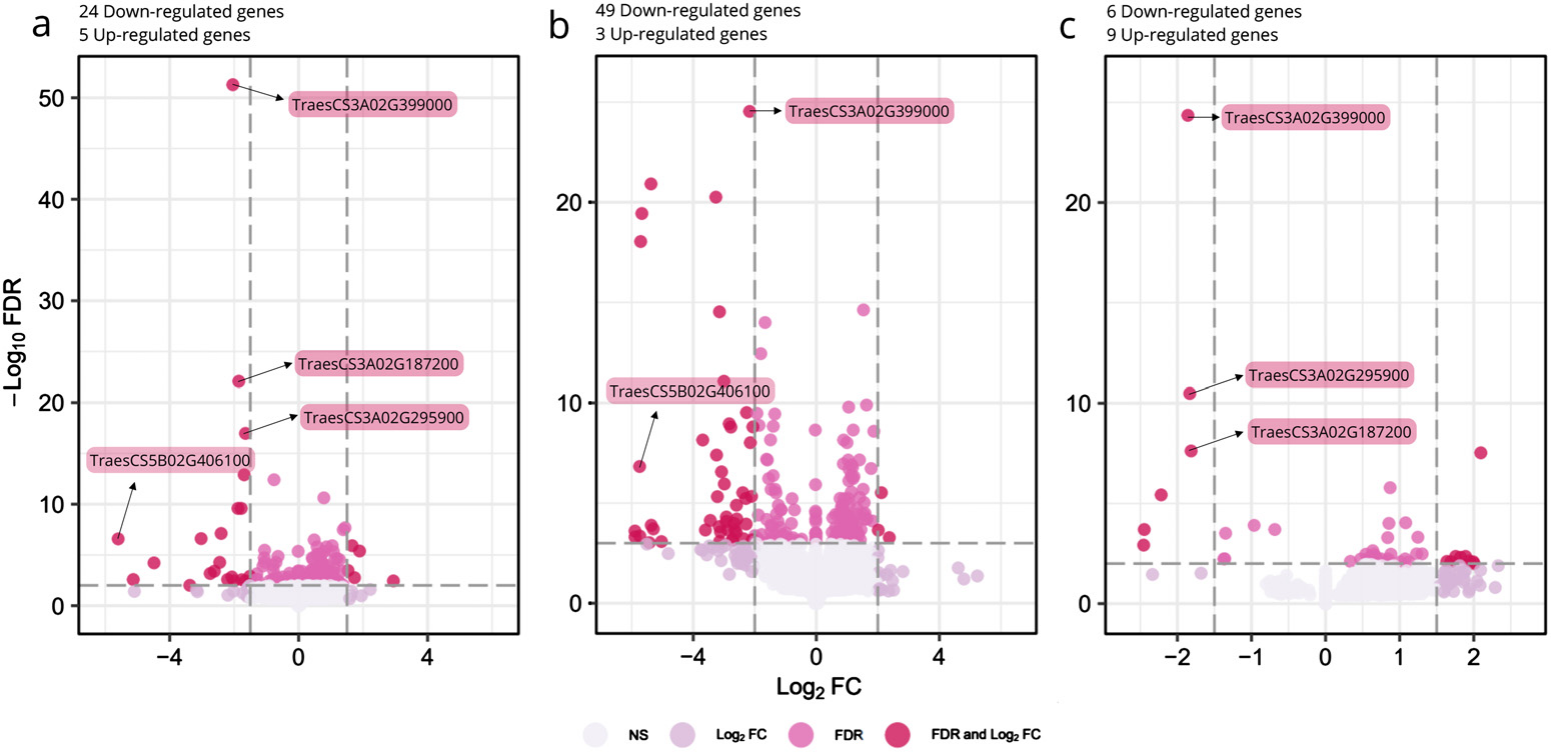
Differentially expressed genes between *srh1* and wild-type, based on RNA-seq of root tissues from BC_2_F_3_ germplasm. **(A)**: Overall primary root tissue, **(B)**: Mature primary root tissue, **(C)**: Tip primary root tissue. For **(A–C)**: the x-axis shows Log_2_ fold change (Log_2_FC) in gene expression, with negative and positive values indicating down-regulation and up-regulation respectively. The y-axis shows the significance, -Log_10_ False Discovery Rate (FDR). Dark red points illustrate individual genes satisfying the Log_2_FC and FDR significance thresholds. Highlighted genes are those with either highly significant FDR or large fold changes. Dashed lines illustrate Log_2_FC and FDR cutoffs.

Using false discovery rate (FDR) ≤ 0.01 and a Log_2_ fold change in gene expression (Log_2_FC) ≥ abs(1.5) (equating to a 2.83 fold change in gene expression), 29 genes were significantly differentially regulated in “combined” (i.e tip + mature zone) *srh1* root tissues relative to wild-type (**Table 2**, **Figure 4A**, **Supplementary Figure S4**. See also **Figure 3B**). Of these, 24 genes were down-regulated (Log_2_F C ≤ -1.5) and 5 were up-regulated (Log_2_FC ≥ 1.5).

**Table 2:** Differentially expressed genes in “combined” root tissues between *srh1* and wild-type.

Wheat Gene ID	Log_2_FC	FDR	Gene Functional Annotation	Known Homolog(s)	Putatively Associated Traits
TraesCS5B02G406100*	-5.5982	2.62E-07	bHLH protein	*AtRSL2, OsRSL9*	Root hair length, drought tolerance, spikelet number, anthocyanin content
*TraesCS1D02G347400*	-5.1297	2.67E-03	Protein kinase (putative)		Grain size, grain length, heterosis, disease resistance
*TraesCS4D02G313800*	-4.4859	6.08E-05	Phosphate transporter protein		Oxidative stress, root number, grain shape, panicle length
*TraesCS2A02G207200*	-3.3756	9.92E-03	GDSL esterase/lipase		
*TraesCS7B02G010700*	-3.1662	2.78E-02	GDSL esterase/lipase	*OsGELP76*	Drought tolerance
*TraesCS2B02G174300*	-3.044	3.95E-04	Peroxidase		Root number, lateral root number
TraesCS5A02G401300*	-3.0248	2.47E-07	bHLH protein	*AtRSL2, OsRSL9*	Root hair length, drought tolerance, spikelet number, anthocyanin content
*TraesCS6A02G030900* (*TaNRT2.4*)	-2.7436	6.74E-04	High affinity nitrate transporter	*AtNRT2*	Lateral root length, lateral root number, nitrate content
*TraesCS6A02G031100* (*TaNRT2.2*)	-2.6138	3.97E-04	High affinity nitrate transporter	*AtNRT2*	Lateral root length, lateral root number, nitrate content
*TraesCS7B02G264400*	-2.537	7.49E-03	Cytokinin oxidase/dehydrogenase	*AtCKX2, AtCKX3, AtCKX4, OsCKX10*	Arsenic concentration, seed size, grain yield, grain shape, plant height, root morphology
*TraesCS4B02G317200*	-2.4487	5.43E-05	Phosphate transporter protein		Oxidative stress, root number, grain shape, panicle length
*TraesCS6B02G044100* (*TaNRT2.1*)	-2.4042	7.64E-08	High affinity nitrate transporter	*AtNRT2*	Lateral root length, lateral root number, nitrate content
*TraesCS6D02G035700* (*TaNRT2.3*)	-2.2051	2.65E-03	High affinity nitrate transporter	*AtNRT2*	Lateral root length, lateral root number, nitrate content
*TraesCS3D02G514400*	-2.0713	1.49E-03	NAD-dependent epimerase/dehydratase	*AtVEP1*	Drought tolerance, grain size, seed size
*TraesCS3A02G399000*	-2.0414	5.18E-52	Calreticulin-3 chaperone	*AtCRT3*	Root hair length, transpiration rate, plant height
*TraesCS2B02G368400*	-2.0044	3.61E-03	Nitrate transporter		
*TraesCS3D02G416800*	-1.882	2.59E-10	Loricrin		
*TraesCS3A02G187200*	-1.8562	8.44E-23	NEDD8-activating enzyme E1 catalytic subunit	*AtECR1*	Days to heading, flowering, maturity, stripe rust resistance
*TraesCS3A02G248000*	-1.7809	2.59E-10	Alpha-amylase	*AtAMY3*	Yield, root morphology, stomatal opening, starch digestibility
*TraesCS4B02G033400*	-1.7632	2.75E-03	Germin-like protein 1	*AtGLP4*	
*TraesCS3A02G434400*	-1.7003	1.27E-13	MADS box transcription factor		Grain weight, grain shape, chlorophyll content, stem elongation
*TraesCS5B02G152400*	-1.6506	3.62E-03	Calcium binding protein	*AtCML37*	Drought tolerance, lignin content
*TraesCS3A02G295900*	-1.65	1.05E-17	Ubiquitin carboxyl-terminal hydrolase (putative)		Days to maturity, leaf senescence
*TraesCS3D02G223600*	-1.5127	1.28E-03	Octicosapeptide/Phox/Bem1p domain containing protein		
*TraesCS7A02G101300*	1.5331	3.40E-04	Cytochrome P450 family protein		
*TraesCS4D02G197500*	1.66889	1.22E-06	Phosphatidylinositol transfer protein		
*TraesCS2D02G442900*	1.73303	1.74E-03	Peptide transporter		
*TraesCS3B02G539500*	1.88995	4.12E-06	Cytochrome P450 family protein		
*TraesCS3B02G424600*	2.93918	3.62E-03	DELLA protein		Chlorophyll content

Negative Log_2_ Fold Change (Log_2_FC) values indicate down-regulated genes in srh1, positive Log_2_FC values indicate up-regulated genes in srh1. Homoeologous wheat genes are indicated: underlined (high affinity nitrate transporters), * (basic helix-loop helix, bHLH). TaNRT2 gene names follow recent nomenclature of Sigalas et al., 2023. Gene prefixes for known orthologs: At: Arabidopsis thaliana, Os: Oryza sativa.

Across “combined” root tissues, the four most significantly down-regulated genes were all located on chromosome 3A (Log_2_FC ≥ -1.6, FDR ≤ 1.27E-13). Of these, *TaCRT3-A* (*TraesCS3A02G399000*, located at 646.1Mb) was the most significantly down-regulated gene between the “combined” root tissues (Log_2_FC -2.04, FDR 5.18E-52) in *srh1* versus wild-type. This pattern was repeated for *TaCRT3-A* in mature and root tip tissues (**Figures 4B, C**). Additional down-regulated genes located on the long arm of chromosome 3A and down-regulated was a putative Ubiquitin carboxyl-terminal hydrolase (*TraesCS3A02G295900*, at 530.2Mb) and a MADS-box transcription factor (*TraesCS3A02G434400*, at 676.2Mb) orthologous to rice gene *OsMADS65*. The most strongly down-regulated (FDR ≤2.62E-03) DEGs were the bHLH gene *TraesCS5B02G406100*, a homolog of the *Arabidopsis* root hair gene *AtRSL2*; a leucine rich protein kinase (*TraesCS1D02G347400*); a phosphate transporter protein (*TraesC S4D02G313800*), and a GDSL esterase/lipase (*TraesCS2A02G207200*) (Log_2_FC -5.60, -5.13, -4.49 and -3.38 respectively).

Five significantly up-regulated genes were identified in *srh1* root tissues, of which four were predominantly upregulated in the *srh1* mature zone (**Supplementary Figure S4**). These include *TraesCS3B02G424600*, predicted to encode a DELLA protein; *TraesCS3B02G539500* and *TraesCS7A02G101300*, predicted to encode Cytochrome P450 oxidoreductases; *TraesCS2D02G442900*, predicted to encode a peptide transporter, and *TraesCS4D02G197500*, predicted to encode a Phosphatidylinositol transfer protein (Log_2_FC 2.94, 1.89, 1.53, 1.73, 1.67 respectively, all with FDR ≤ 1.73E -03, **Table 2**).

We identified 10 DEGs located on Chromosome 3A, and while none of these colocalized with the peak region of the *srh1* locus (596 - 617Mb, BFR ≥ 0.5), the calreticulin-3 chaperone gene *TaCRT3-A* was located within the wider *srh1* interval (584 - 647Mb).

## Discussion

### Root Hair mutants as an entry point for trait improvement

In cereal crop breeding, while much emphasis has been placed on targeting above ground traits for increased crop yield and performance, less focus has been placed historically on root traits. With increasing interest in cereal root phenotypes (Ober et al., 2021), optimization of root hair morphology is likely to play an ever increasing role in ensuring adequate crop nutrition and resilience under future agricultural environments characterized by reduced inputs and the challenges of climate change. To date, the only previously characterized wheat root hair mutant is the *Stumpy* locus on chromosome 7B, which results in long root hairs and very short roots, but only when grown in soil with high levels of (Ca^2+^ (Zeng et al., 2024). In this study, we provide detailed characterization of the first known mutant to display short root hairs in wheat, termed here *srh1*.

### *srh1* is a root hair elongation mutant with altered root growth

Unlike other crop species, little is known about the genetics, genes and gene pathways regulating wheat root hair development. Here, the *srh1* mutant was identified in a mutagenized TILLING population constructed in a spring wheat (cv. Cadenza) background.

While numerous genes controlling the first two stages of root hair development have been described in *Arabidopsis* (epidermal cell fate determination and bulge initiation), to date, none have been identified in the world’s most important cereal crops - rice, maize or wheat (Tsang et al., 2024). In the wheat *srh1* mutant, while most root hairs fail to elongate via tip growth after bulge initiation, some hairs still undergo partial root hair elongation, which is more evident on seminal roots (**Figure 1A**). Notably, the root hair phenotype caused by early termination of tip growth in *srh1* is also found in root hair mutants across different cereal species, including the barley (*Hordeum vulgare* L.) cellulose synthase mutant *hvsclc1* (Gajek et al., 2021) and rice expansin mutant *osexpa17* (ZhiMing et al., 2011). Wheat *srh1* also had longer primary and overall root length compared to wild-type at the seedling stage (**Figures 1M, N**). As we did not observe any differences in trichoblast length between the two genotypes (data not shown), the longer roots in *srh1* are unlikely to be a result of differential cell elongation, or a negative compensatory correlation between root hair and trichoblast length.

### *srh1* candidate genes

Through exome capture and BSA, we localized *srh1* to a chromosomal region of interest (584 - 647Mb), within which a peak region was located between 595 - 617Mb. Within this peak region, seven candidate genes were identified based on filtering for genes with non-synonymous/premature stop mutations, SIFT score and expression in root tissue (**Table 1**). All seven of these genes were found to be expressed in our RNA-seq data, though none were found to be differentially expressed based on the FDR and Log_2_FC criteria used. The amino acid residues of the mutations present in these genes in *srh1* were all conserved by at least 70% across other plant homologs. Below we consider these seven candidate genes with reference to gene expression data in the wheat gene expression atlas (Ramírez-González et al., 2018, **Supplementary Figure S5**) and the wheat single-cell RNA-seq atlas (Zhang et al., 2023).

**Figure 5:**
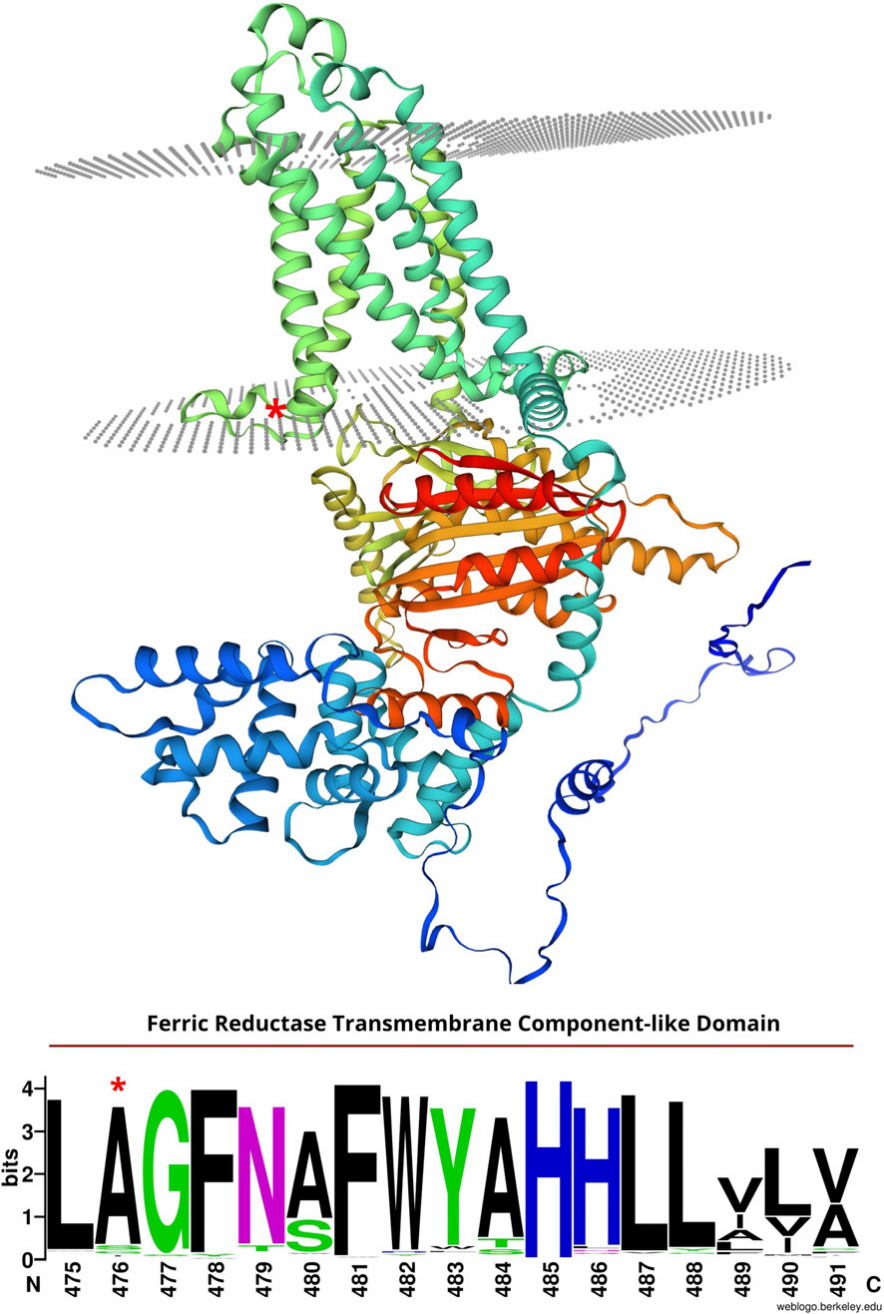
Top: Predicted structure of the *srh1* candidate protein, TaNOX3-A, predicted to encode a respiratory burst oxidase homolog (RBOH). Grey dots illustrate membrane layers. Model predicted using SWISS-MODEL (https://swissmodel.expasy.org/). Bottom: Conservation of amino acid resides 475-491 in TaNOX3-A along with 157 other plant homologs. Letter size illustrates degree of conservation. *TaNOX3-A missense substitution in *srh1*.

Analysis of publicly available single-cell RNA-seq data (Zhang et al., 2023) showed that three of the seven genes are not expressed in root hair cells (protein kinase, *TraesCS3A02G351500*; pumilio-like, *TraesCS3A02G361700*; BEL-1 like homoeodomain, *TraesCS3A02G363900*), and are less likely to encode SRH1. Of the remaining four candidates, *TaNOX3-A* (*TraesCS3A02G354200*) is notable in that it is predicted to encode a respiratory burst oxidase homolog (RBOH) orthologous to known root hair genes in both maize (*ZmRTH5*, Nestler et al., 2014) and rice (*OsNOX3*, Wang et al., 2018). *TaNOX 3-A* is highly expressed in wheat root hair cells relative to all other root tissues (Zhang et al., 2023). Missense mutations in RBOH encoding genes in other cereal species (**Supplementary Figure S1**) result in arrested root hair phenotype. In *Arabidopsis*, ROS production is key for regulating cell growth and root hair development (Foreman et al., 2003; Carol, 2006). Close *Arabidopsis* homologs of *TaNOX3-A* include *RBOHH* and *RBOHJ* (as identified via the ‘Orthologues’ function in Ensembl). *RBOHH* and *RBOHJ* are primarily expressed in mature pollen grains and regulate pollen tube growth - a process identical to how root hairs elongate (Zhou et al., 2020). Mutations in both *RBOHH* and *RBOHJ* result in defective pollen tube growth, as well as a defective root hair phenotype (Zhou et al., 2020). In *ZmRTH5*, a missense mutation resulting in an a.C821Y substitution in the predicted protein encodes the *loss-of-function* allele, whereby *zmrth5* root hairs fail to elongate and are less dense than wild-type maize (Nestler et al., 2014). In the rice *osnox3* mutant, a missense mutation leading to an a.S676N substitution in the predicted protein results in reduced root hair length and density (Wang et al., 2018). RBOHs are membrane bound proteins that produce ROS in root hair tips, which is critical for cell wall extensibility and subsequent root hair initiation and elongation (Nestler et al., 2014). Both amino acid substitutions in *zmrth5* and *osnox3* span NAD binding domains towards the C-termini of their predicted proteins, while the a.A476V substitution in TaNOX3-A spans a ferric reductase transmembrane domain further towards the C-terminus. The TaNOX3-A alanine amino acid residue at position 476 is 100% conserved across wheat homoeologues present in 8 other cultivars with sequenced genomes (International Wheat Genome Sequencing Consortium et al., 2018; Walkowiak et al., 2020) (**Supplementary Figure S6b**), and highly conserved across all cereal orthologues, including the predicted proteins encoded by *OsNOX3*, *ZmRTH5*, barley (*HORVU.MOREX.r3.3HG0303410.1*) and *Brachypodium distachyon* (*BRADI_2g54240v*3). While the TaNOX3-A a.A476V substitution is not predicted to result in a major conformation change (**Supplementary Figure S1**), it’s location in a well conserved ferric reductase transmembrane domain (**Figure 5**) could alter the stability of membrane binding, the ability of the protein to interact with downstream signaling factors, or its ability to produce ROS. Our results indicated ROS production in root hair tips was much lower in *srh1* than wild-type, supporting *TaNXO3-A* as a candidate for *srh1*.

### Insights into the gene pathways disrupted in the *srh1* mutant

Through RNA-seq of primary root tissues of *srh1* and wild-type, and using relatively stringent significance (FDR ≤ 0.01) and gene expression fold change (Log_2_FC ≥ abs(1.5)) criteria, we identified 29 DEGs. Of these, five were notable for their exclusive and high gene expression in the wheat seedling root tip in the gene expression atlas (Zhang et al., 2023), including down-regulated homoeologous bHLH genes in *srh1* tissues (*TraesCS5A02G401300* and *TraesCS5B02G406100*) which show sequence conservation with *RSL* genes controlling root hair elongation in *Arabidopsis* (e.g. Vijayakumar et al., 2016). Previously, increased expression of the wheat *RSL* -like genes *TaRSL2-A* and *TaRSL2-B* on the group 4 chromosomes (most homologous to *Arabidopsis AtRSL4*) have been correlated with longer root hairs that are linked to increased wheat ploidy (Han et al., 2017). Indeed, *RSL* DEG *TraesCS5B02G406100* was the most down-regulated gene identified in the *srh1* mutant. Thus, our results further support the role of the members of the wheat *RSL* gene family in root hair differentiation (**Supplementary Figure S5**). As may be expected in a *loss-of-function* mutant, the majority of DEGs (>80%) were down-regulated (24 out of 29), of which, seven were predicted to encode nitrate or phosphate transporters. Plant root and root hair development is known to be controlled by the interplay between genetic factors and external nutrient concentrations (reviewed by Liu et al., 2020), and previous studies have demonstrated that nitrate transporters are progressively upregulated in root hairs over developmental time (Shanks et al., 2024). Analysis of the wheat gene expression atlas shows that three of the high-affinity nitrate transport genes we identified as DEGs are most highly expressed in the root tissues at the tillering stage, while the fourth is most highly expressed during anthesis (**Supplementary Figure S5**). As such, downregulation of genes involved in these pathways fits with the disrupted *srh1* root hair phenotype.

While it is possible that the hypothetical loss or alteration-of function in TaNOX3-A affects the downstream expression of the down-regulated genes (such as the aforementioned nitrate and phosphate transporters), it may be more likely that the downregulation is primarily associated with the root hair phenotype itself. With shorter root hairs in the *srh1* mutant, less physical space is available on the surface of the root hair membrane for the transporters to be inserted, and thus, lower expression of these genes may be expected.

Of the five DEGs that were upregulated in *srh1*, *TraesCS3B02G424600* (encoding a GRAS-domain DELLA protein) had the highest increase in expression in the mutant compared to wild-type (Log_2_FC 2.94, **Table 2**). Members of this class of DELLA protein are known to act as repressors of signaling by the plant phytohormone gibberellin, and can affect root elongation and radial patterning (Hirsch and Oldroyd, 2009). These GRAS encoding genes also promote beneficial root-fungal associations, including in the cereal species rice and barley, highlighting further possible mechanisms for the interaction between root structure and function (Li et al., 2022).

Of all DEGs identified in *srh1*, *TaCRT3-A* was most significantly down-regulated in the mutant, in both mature root and root tip root tissues (**Supplementary Text 1**). Located at 646Mb on chromosome 3A, while *TaCRT3-A* is not within the peak of the *srh1* locus as identified via BSA (595 - 617Mb), it is within the wider *srh1* interval (584 - 647Mb). *TaCRT3-A* is predicted to encode a wheat calreticulin - an endoplasmic reticulum bound chaperone involved in calcium signaling. Calreticulin functions in plants are associated with tip growth (Suwińska et al., 2017), drought stress (Jia et al., 2008) and plant height (Jin et al., 2009). Importantly, calcium signaling is known to be a crucial process in controlling plant root hair development. For example, the calcium protein kinase ZmCPK9 in maize is responsible for root hair elongation by maintaining a high Ca^2+^ concentration at the root hair apex (Zhang et al., 2020). The formation of intracellular calcium gradients in a root hair also aids the establishment of cell polarity (Brost et al., 2019). Indeed, this class of gene has also been shown to affect root hair development in *Arabidopsis* (Vu et al., 2017). The recent finding of a wheat root hair mutant that only exhibits the long root hair phenotype under high soil Ca^2+^ levels (Zeng et al., 2024), further highlights the role of calcium signaling in the control of wheat root hair growth.

Collectively, our DEG analysis indicates that disrupting root hair development through *srh1* impacts expression of many genes with a range of putative functions, several of which are linked to root hair development in *Arabidopsis* and other plant species through either direct or indirect mechanisms. In the near future, we aim to explore how the *srh1* mutant performs in the field in contrast to the wild-type, and subsequently, quantify the effect of root hairs on wheat field performance.

## Conclusions

Wheat root hairs are involved in numerous root/soil interactions. Here, we describe the first mutant repressing root hair elongation in wheat, providing an entry point into the genes and gene networks controlling wheat root hair development and function. The ability to understand the genetic control of their development, structure, and function, and how this interacts with the environment, will aid the design and development of new, better performing, resilient wheat varieties to support food production under future agricultural scenarios.

## Data Availability

The data presented in the study are deposited in Sequence Read Archive (SRA) under the project number PRJNA1170241.

## Supplementary material

Supplementary Figure 1 Impact of TILLING mutations on the predicted proteins of *srh1* candidate genes. Predicted protein alignment of the missense substitutions between wheat *TaNOX3-A*, rice *OsNOX3* and maize *ZmRTH5* and other plant homologs. **(A)**: *srh1* A >V, **(B)**: *osnox3* S >N, **(C)**: *zmrth5* C >Y. Residues in red indicate mutation position. **(D)**: SWISS-MODEL (https://swissmodel.expasy.org/) protein model of TaCRT3-A, highlighting the TILLING mutation in the *srh1* donor line Cadenza1714 that results in a stop codon at amino acid residue 359 (colored red in the magnified section, and highlighting the disruption of numerous hydrogen bond, dashed lines, with adjacent amino acid residues) and the resulting truncated region (highlighted in green).

Supplementary Figure 2 Exome capture sequencing gene (including flanks of +/- 50% of gene lengths up and downstream of each gene) depths in bulked populations of **(A)** Cadenza wild-type and **(B)** *srh1* mutant BC_1_F_3_ lines. To remove noise seen by spuriously mapped reads, we introduced a lower depth cutoff of 3 and an upper limit of 200. Turquoise lines represent 10*^th^* and 90*^th^* depth percentiles, respectively. Orange and red lines illustrate median and mode depths, respectively, following the removal of depths<3 and >200. For bulked segregant analysis (BSA), lower and upper depth cutoffs were established at 5 and 200, respectively.

Supplementary Figure 3 Amino acid conservation in the predicted proteins of the seven candidate genes identified at the *srh1* genetic locus.

Supplementary Figure 4 Heatmap of differentially expressed genes (DEGs) in seminal root tissues of three day old wild-type versus *srh1* mutant BC_2_F_3_ segregants, identified in this study via RNA-seq. WM = Wild-type Mature Tissues, WT = Wild-type Tip Tissues, MM = Mutant Mature Tissues, MT = Mutant Tip Tissues. Expression in each of the three biological replicates assayed per tissue type are shown.

Supplementary Figure 5 Heatmap illustrating expression of differentially expressed genes (DEGs) identified by RNA-seq in *srh1* versus wild-type. Expression is shown in key tissues across varying growth stages, normalized across each row via a Z-score. Blues indicate higher relative expression of a gene in a particular tissue; reds indicate lower relative expression across the same tissue. Tissues color coded based on growth stage, root tissues underlined. RNAseq expression data from cv. Azhurnaya spring wheat (Wheat eFP browser, Ramírez-González et al., 2018).

Supplementary Figure 6 TaNOX3-A sequences. **(A)**: Sanger sequencing of the mutation in *TaNOX3-A*. 1^st^ and 3^rd^ traces illustrate the genomic sequence of the mutant, 2^nd^ and 4^th^ traces illustrate the genomic sequence of the wild-type. The C >T substitution is clearly visible at sequence position 734 (marked by a red asterisk). **(B)**: Alignment of TaNOX3-A homoeologues from 8 other sequenced wheat genomes (International Wheat Genome Sequencing Consortium et al., 2018; Walkowiak et al., 2020) and orthologues (in rice and maize) within the region spanning the *srh1* mutation. Homoeologues from the wheat reference genome of cv. Chinese Spring are outlined via a black box. The position of the A > V amino acid substitution is marked by a red asterisk. Region shown via Sanger sequencing in **(A)** is correspondingly annotated on **(B)**.

Supplementary Figure 7 Seven day old BC_2_F_4_ *srh1* mutant and wild-type seedling root hair phenotype in soil. **(A-C)**: Wild-type, **(D-F)**: Mutant. Root hairs are clearly visible in the wild-type, and are either absent or vestigial in the mutant.
