# Supplementary figures and images for: A novel root hair mutant, srh1, affects root hair elongation and reactive oxygen species levels in wheat
Source: Front Plant Sci. 2024 Oct 30;15:1490502. doi: 10.3389/fpls.2024.1490502 (PMC11557487; doi:10.3389/fpls.2024.1490502)

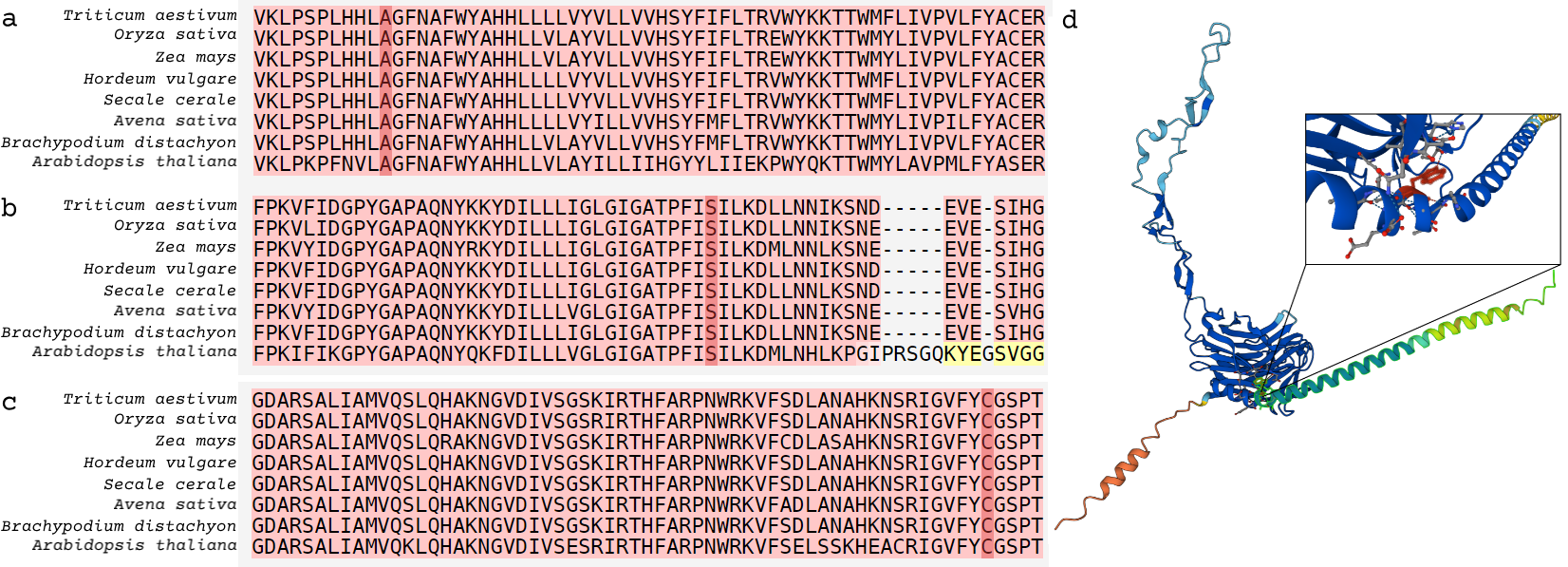

Supplement: Supplementary Figure 1 — Impact of TILLING mutations on the predicted proteins of srh1 candidate genes. Predicted protein alignment of the missense substitutions between wheat TaNOX3-A, rice OsNOX3 and maize ZmRTH5 and other plant homologs. (A): srh1 A >V, (B): osnox3 S >N, (C): zmrth5 C >Y. Residues in red indicate mutation position. (D): SWISS-MODEL (https://swissmodel.expasy.org/) protein model of TaCRT3-A, highlighting the TILLING mutation in the srh1 donor line Cadenza1714 that results in a stop codon at amino acid residue 359 (colored red in the magnified section, and highlighting the disruption of numerous hydrogen bond, dashed lines, with adjacent amino acid residues) and the resulting truncated region (highlighted in green). [file Image1.png]

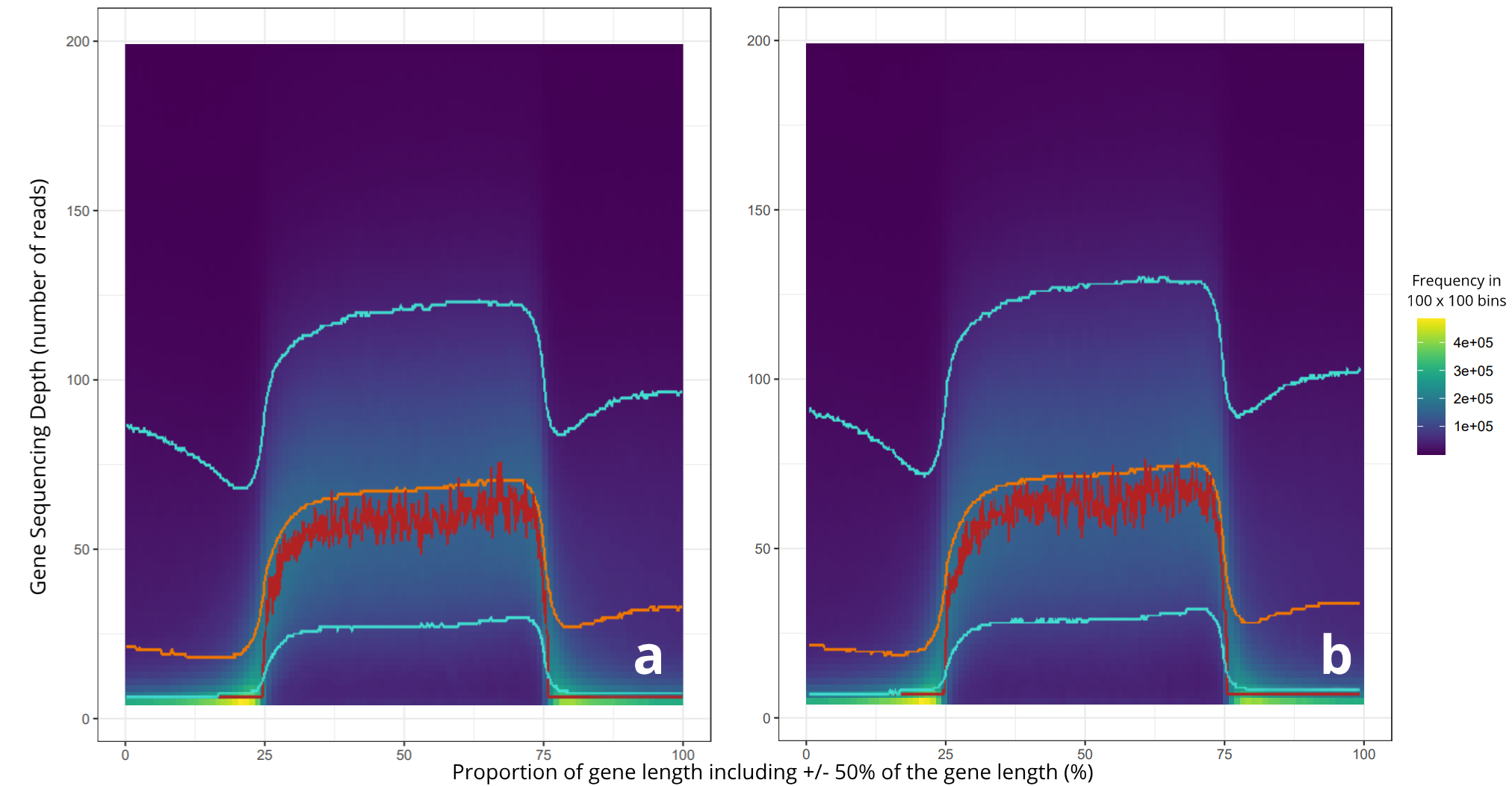

Supplement: Supplementary Figure 2 — Exome capture sequencing gene (including flanks of +/- 50% of gene lengths up and downstream of each gene) depths in bulked populations of (A) Cadenza wild-type and (B) srh1 mutant BC1F3 lines. To remove noise seen by spuriously mapped reads, we introduced a lower depth cutoff of 3 and an upper limit of 200. Turquoise lines represent 10 th and 90 th depth percentiles, respectively. Orange and red lines illustrate median and mode depths, respectively, following the removal of depths<3 and >200. For bulked segregant analysis (BSA), lower and upper depth cutoffs were established at 5 and 200, respectively. [file Image2.png]

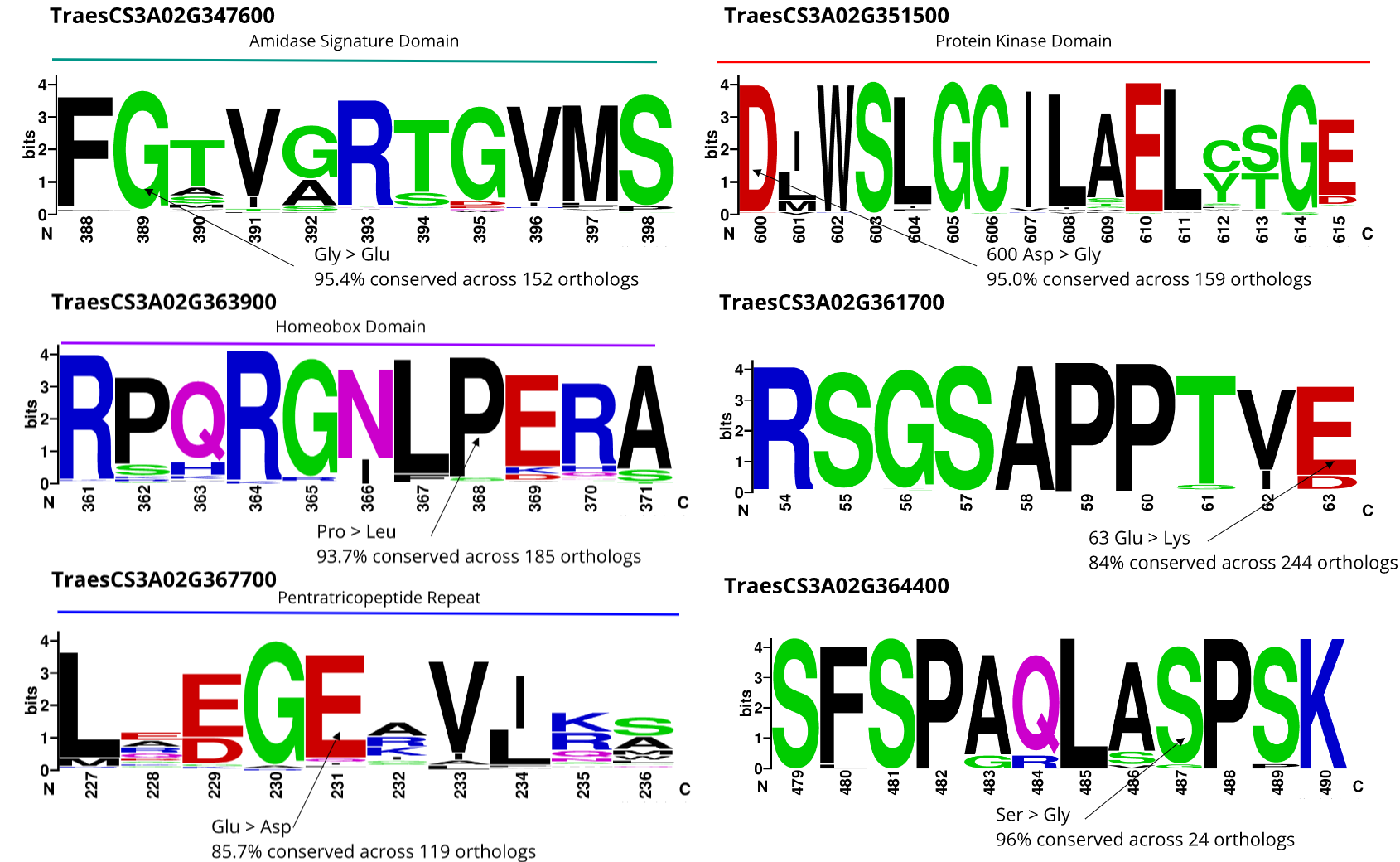

Supplement: Supplementary Figure 3 — Amino acid conservation in the predicted proteins of the seven candidate genes identified at the srh1 genetic locus. [file Image3.png]

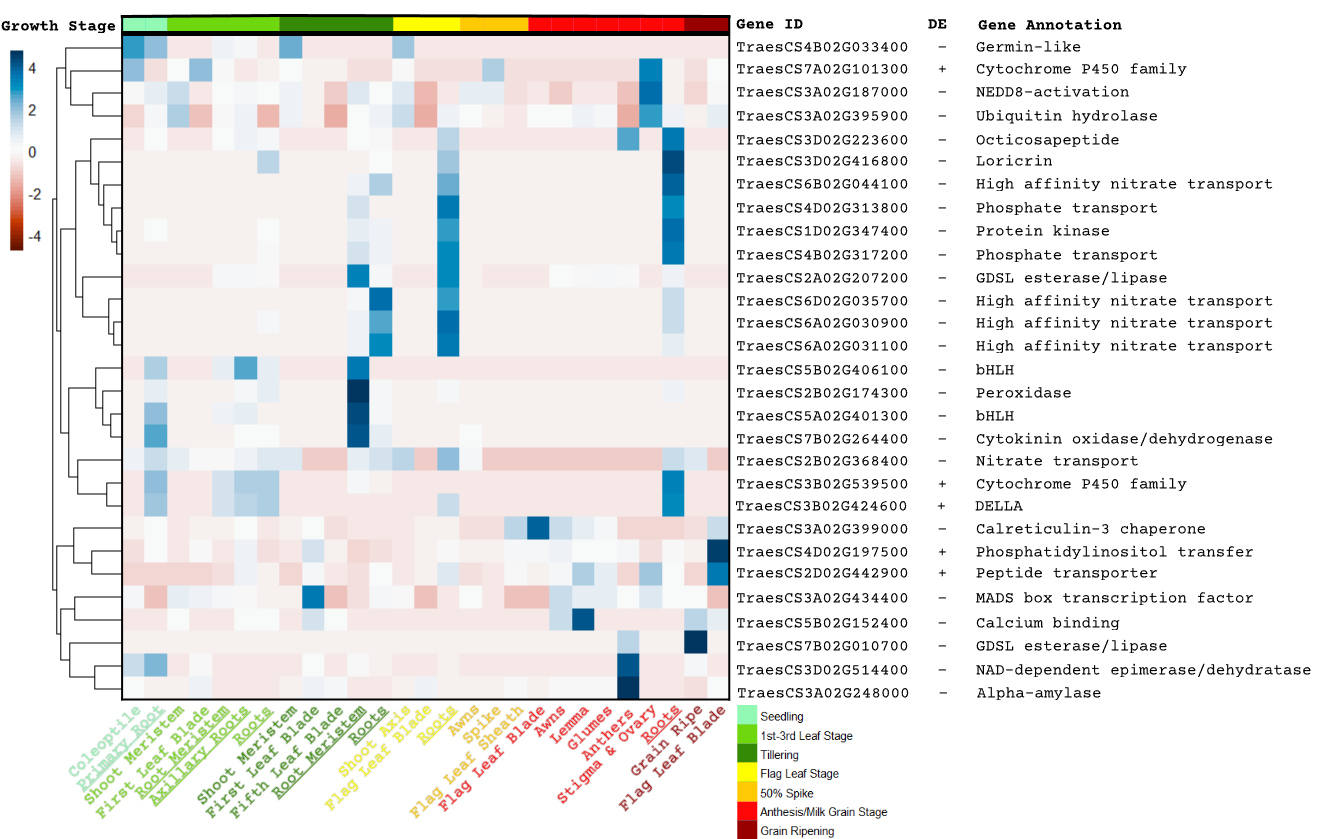

Supplement: Supplementary Figure 4 — Heatmap of differentially expressed genes (DEGs) in seminal root tissues of three day old wild-type versus srh1 mutant BC2F3 segregants, identified in this study via RNA-seq. WM = Wild-type Mature Tissues, WT = Wild-type Tip Tissues, MM = Mutant Mature Tissues, MT = Mutant Tip Tissues. Expression in each of the three biological replicates assayed per tissue type are shown. [file Image4.png]

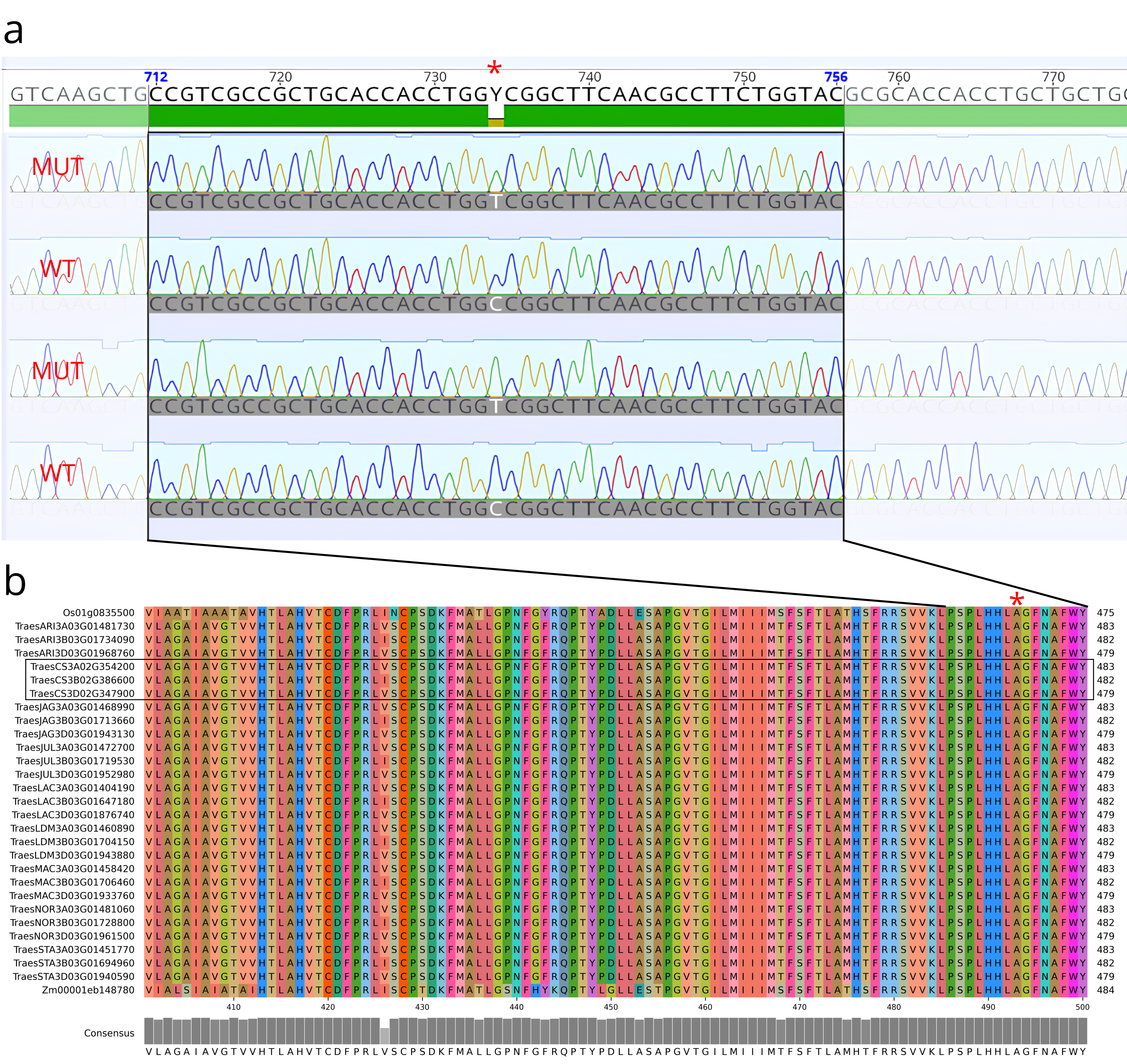

Supplement: Supplementary Figure 5 — Heatmap illustrating expression of differentially expressed genes (DEGs) identified by RNA-seq in srh1 versus wild-type. Expression is shown in key tissues across varying growth stages, normalized across each row via a Z-score. Blues indicate higher relative expression of a gene in a particular tissue; reds indicate lower relative expression across the same tissue. Tissues color coded based on growth stage, root tissues underlined. RNAseq expression data from cv. Azhurnaya spring wheat (Wheat eFP browser, Ramírez-González et al., 2018). [file Image5.png]

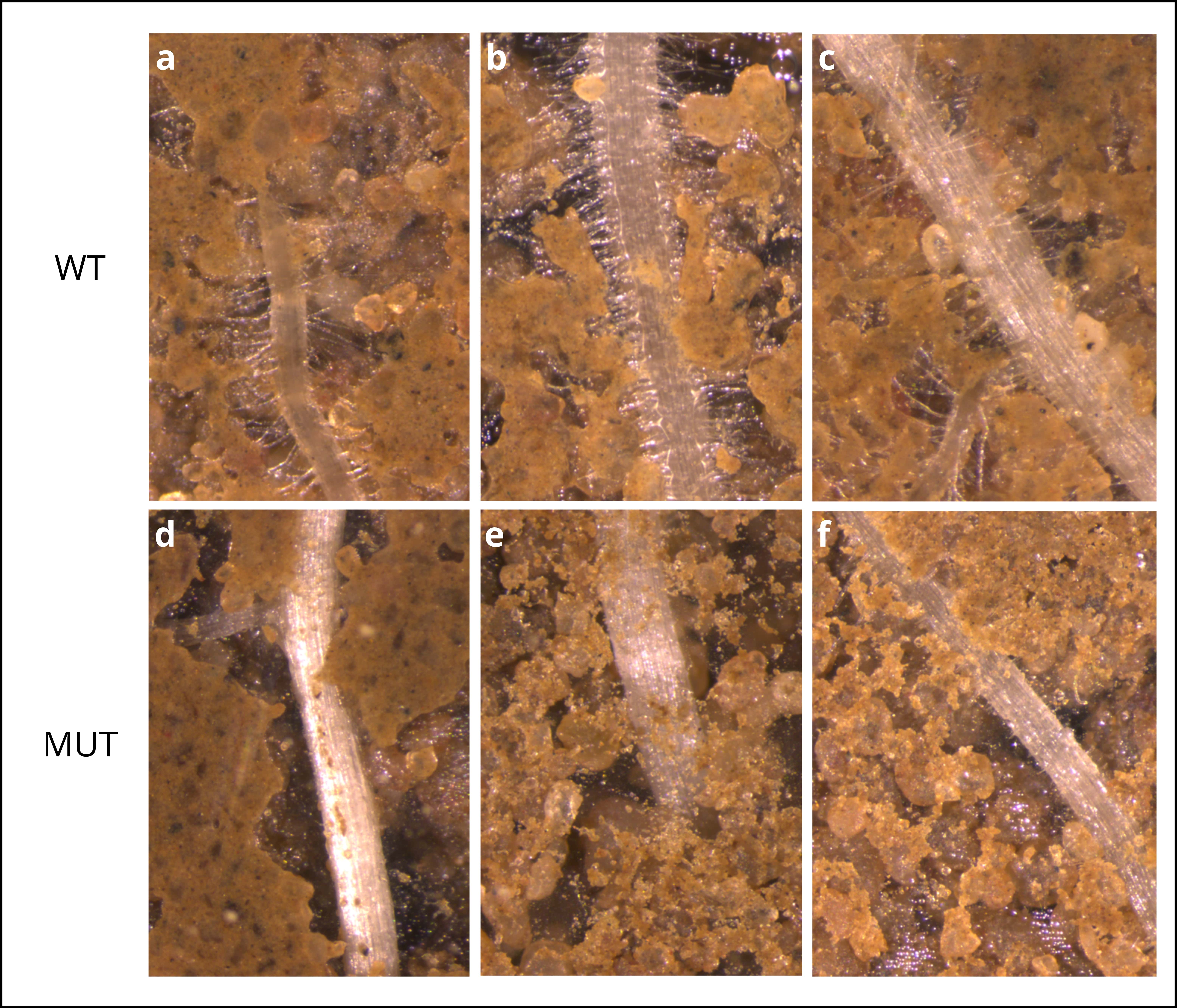

Supplement: Supplementary Figure 6 — TaNOX3-A sequences. (A): Sanger sequencing of the mutation in TaNOX3-A. 1st and 3rd traces illustrate the genomic sequence of the mutant, 2nd and 4th traces illustrate the genomic sequence of the wild-type. The C >T substitution is clearly visible at sequence position 734 (marked by a red asterisk). (B): Alignment of TaNOX3-A homoeologues from 8 other sequenced wheat genomes (International Wheat Genome Sequencing Consortium et al., 2018; Walkowiak et al., 2020) and orthologues (in rice and maize) within the region spanning the srh1 mutation. Homoeologues from the wheat reference genome of cv. Chinese Spring are outlined via a black box. The position of the A > V amino acid substitution is marked by a red asterisk. Region shown via Sanger sequencing in (A) is correspondingly annotated on (B). [file Image6.jpeg]

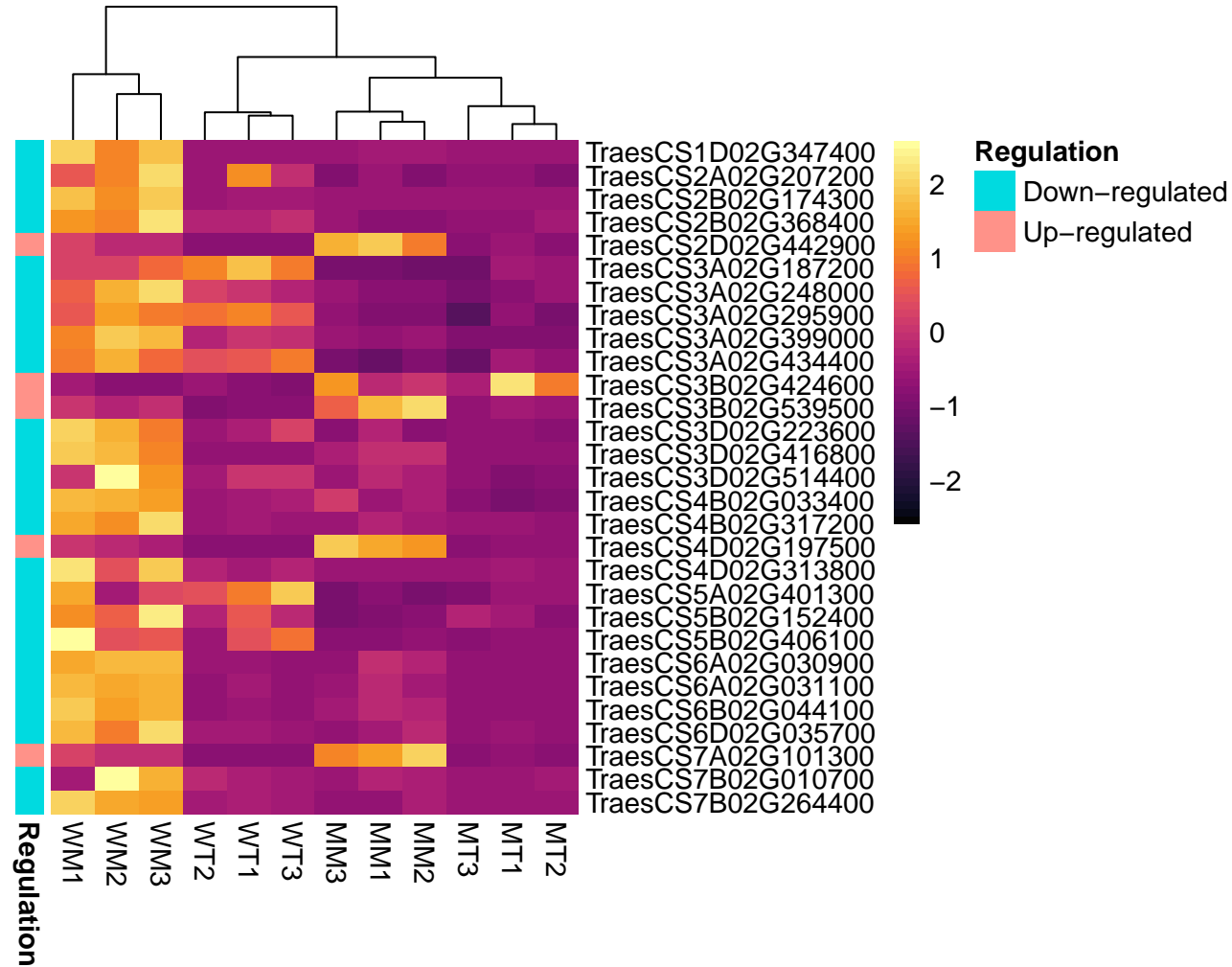

Supplement: Supplementary Figure 7 — Seven day old BC2F4 srh1 mutant and wild-type seedling root hair phenotype in soil. (A-C): Wild-type, (D-F): Mutant. Root hairs are clearly visible in the wild-type, and are either absent or vestigial in the mutant. [file DataSheet1.pdf]
